# “A Dangerous Black Box:” Idiopathic Hemophagocytic Lymphohistiocytosis in Adult Patients—A Case Report and Review of the Literature

**DOI:** 10.1155/2022/5867129

**Published:** 2022-12-03

**Authors:** Nada Agbariah, Javier Sanz, Alicia Rovó

**Affiliations:** ^1^Department of Hematology and Central Hematology Laboratory, Inselspital, Bern University Hospital, University of Bern, Bern, Switzerland; ^2^Department of Human Genetics, Inselspital University Hospital Bern, Bern, Switzerland

## Abstract

Hemophagocytic lymphohistiocytosis (HLH) is a rare potentially life-threatening condition characterized by aberrant inflammation that can be related to genetic or sporadic forms. In both forms, triggering factors may be involved. Early detection of the underlying cause is crucial for therapeutic decision, while early intervention might be associated with better outcomes. The largest descriptions in the literature on HLH refer to pediatric cases. Adolescents and adults may also be affected, but there is scarce evidence regarding their diagnosis and management. We describe here the case of a 68-year-old Swiss woman with HLH, in whom an extensive search for underlying causes was performed, but neither trigger nor pathogenic variant was found. An early intervention first with dexamethasone and later with cyclosporine was performed. The patient showed a favorable response and did not require further hospitalization; however, one year after diagnosis, it was not possible to suspend cyclosporine due to recurrence of laboratory inflammation signs by drug tapering. The occurrence of HLH idiopathic forms represents a challenge; failure to identify the underlying triggering cause generates uncertainty, endless diagnostic investigations, and consequently additional delays in the treatment. This manuscript addresses the difficulties on this issue.

## 1. Introduction

Hemophagocytic lymphohistiocytosis (HLH) is a rare and potentially life-threatening condition characterized by a cytokine storm that is provoked by aberrant activation of macrophages and T cytotoxic cells [1, 2]. Clinically, HLH patients present with fever, splenomegaly, and a sepsis-like syndrome. The laboratory findings are hyperferritinemia, bicytopenia or pancytopenia, activation of the coagulation cascade, hypertriglyceridemia, often hepatitis, and multiorgan dysfunction [3–5]. Pathophysiology is complex: key factors are the presence of predisposing immunodeficiency and significant immune activation [6]. Although sepsis can trigger HLH, it is important to identify underlying associated HLH as soon as possible, because immunosuppressive therapy is required to suppress the cytokine storm of HLH, a strategy that is not present systematically in sepsis guidelines [7, 8].

The mortality of HLH is highly dependent on provoking conditions and lies between 20% and 88% [9–11]. There are two forms of HLH: the primary/genetic one, more frequent in children, is caused by pathogenic variants in genes involved in lymphocytic cytotoxicity and immune regulation. The secondary form, more frequent in teenagers and adults, is associated with different medical conditions, such as infections with Epstein–Barr virus (EBV) or cytomegalovirus (CMV), malignancy, and rheumatologic diseases and more recent cases reported due to SARS-CoV-2 infection or vaccination or after chimeric antigen receptor T-cell (CART) therapy infusions among others [6, 12–17]. Furthermore, in a variable number of patients, despite extensive investigations, no trigger has been found to explain HLH and they will be considered as idiopathic forms. The latter represents a particular challenge since physicians often might insist on endless diagnosis searches that could lead to an unacceptable delay in the treatment.

HLH is a condition better known in children than in adults [1], although awareness regarding HLH in the adult population is increasing. The incidence of HLH in the pediatric population is 1 in 300.000 [12, 13, 18], whereas the real incidence of HLH in the adult population remains unknown; however, in the last 10 years, the number of cases reported in the literature in the adult population has significantly increased [4, 19, 20]. Diagnostic tools such as HLH-2004 [21] are based on pediatric populations, and despite being applied to adult patients, their interpretation must be performed with caution [22]. In contrast, the HLH-score (HScore), which was published in 2014, is based on an adult population [23] and relies on the summation of points assigned for 9 variables (immunosuppression, fever, organomegaly, triglyceride, ferritin, glutamic oxaloacetic transaminase, fibrinogen, cytopenia, and hemophagocytosis features in bone marrow aspirates), which correlate with the probability of having HLH. The Hscore ranges from 0 to 337, giving a probability of having HLH ranging from <1% to ≥99% [23].

There are recommendations for the treatment of HLH in pediatric patients, and for example, HLH-2004 [24], an established treatment protocol for adult HLH patients, is however missing. Thus, treatment of adult HLH patients follows existing recommendations for pediatric patients with a variety of nonstandard adaptations. It is common practice to start the treatment with glucocorticoids and another immunosuppressive drug, such as cyclosporine. The use of etoposide, as recommended by HLH-2004, despite being frequently used, is still discussed in adult patients. Allogenic stem cell transplantation has rarely been reported in adults with secondary HLH [25].

A literature search was performed in PubMed using MeSH terms (performed on January 23^rd^, 2022; HLH adult). Among the 1,192 results, 28 cohorts including more than 10 adult patients published from 2010 were identified and evaluated (Table 1).

## 2. Case Presentation

We report the case of a 68-year-old Swiss woman who presented to a peripheral hospital in Switzerland after consulting her general doctor about loss of weight (8 kg) in the last month, a maculopapular rash, fatigue, loss of appetite, palpitations, headaches, and cough for 2 weeks. The general doctor assigned her to the peripheral hospital after measuring 39.5°C fever and noticing very high liver parameters in the blood test.

She had a history of pericarditis at the age of 65 years, and paroxysmal atrial fibrillation and depression were also noted.

The family history was negative for hematological diseases.

The laboratory workup at admission showed normocytic, normochromic nonregenerative moderate anemia, mild thrombocytopenia, high D-dimer, slightly lower fibrinogen, liver insufficiency, and high triglycerides. The ferritin level increased (7,069 *μ*g/L, reference value: 10–120 *μ*g/L). An ultrasound scan of the abdomen showed splenomegaly (12.5 cm). The SARS-CoV-2 polymerase chain reaction (PCR) test, chest X-ray, lumbar puncture, blood culture, serology for EBV, CMV, human immunodeficiency virus (HIV), hepatitis A, B, C, and E, *Coxiella*, parvovirus B19, and *Bartonella*, and search for *Mycobacterium tuberculosis* (QuantiFERON test) were negative. After one week of hospitalization, her clinical condition deteriorated, and she was transferred to our center with the suspicion of HLH. At admission, the patient presented with markedly reduced general conditions; she was febrile (38.6°C), hemodynamically compensated, her BMI was 18.5 kg/m^2^, and the laboratory tests showed a progressive increase in CRP up to 190 mg/l, hypoosmolar hyponatremia, increased liver values, coagulopathy, and pancytopenia (Table 2). An HScore for reactive HLH was calculated, showing 209/337 points with an estimated probability for HLH of 93% (Table 3). Therefore, therapy with dexamethasone 15 mg/day was immediately started. Additional investigations were performed, including transthoracic echocardiography, computed tomography (CT) of the chest, abdomen, and pelvis, and a biopsy of the liver, skin, and bone marrow; however, none of these examinations contributed to the identification of an underlying trigger for HLH. The bone marrow showed a large number of macrophages but did not show any sign of hemophagocytosis. Furthermore, a rheumatologic disease could be excluded based on negative screening for autoantibodies (antinuclear antibodies, Hep-2, SsA, PR3 antineutrophil cytoplasmic antibodies, and myeloperoxidase antineutrophil cytoplasmic antibodies) and missing related symptoms. After 1 week of treatment, slight clinical and laboratory improvements allowed doctors to start tapering dexamethasone, and the patient was discharged home after 4 weeks of hospitalization.

Four days later, she presented to her scheduled control in the outpatient clinic of hematology with worsened fatigue. The laboratory workup showed an increase of ferritin to 49′036 mcg/L while still being under 5 mg dexamethasone/day. We therefore increased the dexamethasone dose to 16 mg/day and started with cyclosporine, with a trough level goal between 100 and 200 ng/ml. Because up to that point no cause of HLH could be identified, the investigation was completed with positron emission tomography-computed tomography (PEC-CT), which showed intensive metabolic activity behind the right patella and in the ascending and sigmoid colon. Gastroscopy and colonoscopy performed afterward excluded gastrointestinal neoplasia and local inflammatory diseases. Additionally, leg magnetic resonance imaging (MRI) was performed, which suggested pigmented villonodular synovitis (PVNS), a benign entity unlikely to cause HLH, and thus considered an incidental finding.

Only after 3 months from the manifestation of symptoms, the patient reported a significant improvement in her general condition, including regaining appetite and weight.

Furthermore, we performed a genetic analysis searching for variants in genes related to primary HLH (perforin 1 (*PRF1*), syntaxin 11 (*STX11*), syntaxin binding protein 2 (*STXBP2*), and *UNC13D*), revealing no pathogenic variants.

The diagnostic workup is summarized in Table 4.

Vitamins and substrates (folic acid, iron, zinc, and vitamin B12) were administrated to sustain hematopoiesis.

Six months after the diagnosis, the patient was completely back to her routine life with no symptoms and completely normalized laboratory findings (Table 2). Slow tapering to discontinue cyclosporine was performed, but immediately before planned drug withdrawal, laboratory signs of inflammation with increased liver values at a 9-monthfollow-up were observed, and a low dose of cyclosporine was reinstalled. Currently, one year after the diagnosis, the patient is doing well, and tapering of cyclosporine is still ongoing.

## 3. Discussion/Conclusion

Several causes have been described as possible triggers of HLH in adult patients (Table 4). Nevertheless, the case presented here did not show any underlying trigger despite an exhaustive search.

The most important contribution of bone marrow investigations in HLH is the diagnosis or exclusion of hematological diseases. In the bone marrow cytology and biopsy of the case reported here, there was no evidence of lymphoma or other hematological diseases, which may have triggered HLH, and there was also no evidence of hemophagocytosis. Indeed, hemophagocytosis in the bone marrow is reported in just 25% of HLH cases [26]. It is important to note that finding a hemophagocytosis phenomenon in the bone marrow is neither specific nor sensitive for HLH [27]. Furthermore, in our case, a skin biopsy was performed to rule out Sézary syndrome due to the presence of maculopapular exanthema erythematosus of unknown origin. Likewise, due to the persistence of liver test abnormalities, a liver biopsy was also performed. The results of both biopsies did not show lymphoma or other relevant findings, and in both samples, no hemophagocytosis phenomenon was observed.

Performing PET-CT on HLH patients contributes to the diagnosis of lymphoma and other malignant or inflammatory diseases [28–30].

After excluding a secondary cause of HLH in our patient and despite the low probability due to age, as well as the lack of positive family history, we considered the possibility of primary/genetic HLH. This form is better known in children and often associated with other abnormalities such as immunodeficiency or albinism, such as in Griscelli syndrome [31]. Panel sequencing of HLH-associated genes or exome sequencing have made a great deal of contribution to the diagnosis of congenital forms, particularly in children. In fact, with these investigations, molecular explanation could likely be found in 58% of the studied pediatric patients even in the absence of familial syndromes [32]. The most common pathogenic variants reported in 14% of the cases associated with HLH in adults were identified in *PRF1*, *STXBP2*, and *UNC13D* [33]. In our patient, the analysis of these genes was negative.

With the first suspicion of HLH, despite not knowing the triggering cause, an early intervention first with dexamethasone and later with cyclosporine was performed, as suggested by other authors [1, 5]. The patient initially responded well to glucocorticoid monotherapy, but while tapering at one month (Table 2), she had a relapse. A careful glucocorticoid tapering method combined with another immunosuppressive (cyclosporine) drug was afterward successful in this case.

In the literature review, we found 28 case series or studies published after 2010 based on the adult population [11, 26, 28, 30, 34–57]. Cohorts were collected from different countries and reported on a number of patients, from 12 [46] to 264 [38]. A meta-analysis [11] analyzing 2,197 patients was also included. We found that the most common trigger reported for secondary HLH in adults was malignancy, both solid cancer and hematological diseases, with a weighted average of 50% (from 0% to 72%). The second most common trigger was infection with 39% (from 0% to 76%) and autoimmune diseases with 10% (0% to 58.3%). Idiopathic HLH was reported in 24/28 studies, and the weighted average was 9.4% (0% to 50%). The different observed prevalence of triggers may be explained by the differences in the median age of the studied population. Furthermore, not all centers had the same possibility and easy access to diagnostic tools such as MRI or PET-CT and therefore were not included in publications. This diversification in patients and used diagnostic methods may partially contribute to the variability of the prevalence of idiopathic HLH.

Regarding the mortality, some studies reported just the 30-day mortality, others the mortality during the hospitalization or for the whole follow-up. For this analysis, we focused on the 30-day mortality, which was 38%, and on the overall mortality, which was 53%. In addition, mortality may be influenced by different factors, such as the prevalence of different underlying diseases, comorbidities, and age of the observed population, as well as the experience and resources of the center in diagnosing and treating HLH rapidly.

HLH is a life-threatening condition and requires rapid diagnosis and therapy. Unknown origin of HLH may be frustrating for both physicians and patients. According to our experience, it is very important to perform a complete diagnostic test, including tissue biopsy, different imaging studies, and genetic analysis whenever possible. All possible triggers should be searched because specific treatment of the underlying “trigger” disease may play a key role in management [5]. Every diagnostic and therapeutic step must be explained clearly to the patient to keep high compliance, which is essential in this process that may last several months in some cases. Our patient was very consistent with the prescribed medications and the appointments in our outpatient clinic, and she also had great support from her husband. They understood how dangerous her condition was; nowadays, they are happy with the outcome and the treatment received, and the patient is still under follow-up. A close follow-up is recommended after the first episode of idiopathic HLH to allow early diagnosis of an eventual relapse.

With currently available diagnostic methods, idiopathic forms of HLH still exist. Considering the high reported rate of mortality in many series, it is important to emphasize that in seriously affected patients, the lack of a trigger should not delay the initiation of immunosuppressive treatment, which should be started as soon as possible and should be complemented by the search for underlying causes.

We can conclude that despite the recent contributions of some publications, there is clearly a deficit in the understanding of HLH in adults. Due to the rarity of its presentation, only joint efforts of many centers investigating this disease in a prospective way will better identify its epidemiological, diagnostic, and therapeutic aspects, allowing for tailored management of HLH adults in the future.

## Figures and Tables

**Table 1:** Case series and cohorts of HLH in adult patients. In some studies, more than a possible cause for HLH was identified.

Study	Number of patients	Malignancy	Hematological disease	Primary immunodeficiency	Infection	Autoimmune disease	Post-transplant	Other	Idiopathic	30-daymortality	Overall mortality
Ahn et al., 2010	30	4 (13.3%)	—	—	18 (60%)	1 (3.3)	—	—	7 (23.3%)	14 (46.7%)^+^	NA
Tseng et al., 2010	96	61 (63.5%)	—	—	32 (33.3%)	3 (3.2%)	—	—	—	NA	60 (63%)
Shabbir et al., 2010	18	4 (22%)	1 (6%)	—	5 (28%)	2 (11%)	3 (16%)	—	3 (17%)	5 (27%)	12 (66.7%)
Park et al., 2012	23	Excluded	—	—	17 (74%)	—	—	—	6 (23%)	NA	17 (73.9%)
Kim et al., 2013	14	14 (42.8%)	—	—	14 (14.3%)	14 (14.3%)	—	—	14 (28.6%)	NA	5 (35.7%)
Parikh et al. 2014	62	32 (52%)			21 (34%)	5 (8%)	—	—	4 (6%)	27 (44%)	41 (66%)
Ramos et al., 2014^++^	2197	1047 (47.66%)	—	—	1108 (50.4%)	276 (12.6%)	—	184 (8.4%)	81 (3.7%)	NA	1109 (41%)
Riviere et al., 2014	162	95 (59.9%)	—	—	40 (24.7%)	5 (3.1%)	—	-	14 (8.6%)	68 (42%)	33 (20%)
Barba et al., 2015	71	21 (30%)	—	—	20 (28%)	13 (13%)	—	—	18 (26%)	40 (41%)	68 (70%)
Li et al., 2015	85	23 (27.1%)	—	—	29 (34.1%)	6 (7.1%)	—	—	27 (31.8%)	NA	39 (65%)^+++^
Schram et al., 2015	68	33 (49%)	—	—	22 (33%)	19 (28%)	—	—	15 (22%)	14 (21%)	46 (69%)
Oto et al., 2015	34	6 (17.6%)	—	—	4 (11.8%)	3 (8.8%)	—	4 (11.7%)	17 (50%)	NA	11 (32.4%)
Li et al., 2016	103	49 (47.6%)	—	—	24 (23.3)	14 (13.6%)	—	—	24 (23.3%)	NA	77 (74.8%)
Lim et al., 2016	264	170 (64.4%)	—	—	48 (18.2%)	15 (23.4%)	—	—	31 (11.7%)	NA	133 (50.37%)
Yuan et al., 2016	45	28 (62.2%)	—	—	10 (22.2%)	—	—	—	—	NA	NA
Zheng et al., 2016	43	31 (72%)	—	—	3 (7%)	3 (7%)	—	—	6 (14%)	NA	31 (72%)
Arslan et al., 2018	26	3 (11.5%)	—	—	9 (34.6%)	4(15.4%)	—	—	10 (38.5%)	NA	13 (50%)
Brito-Zeron et al., 2018	151	48 (31.8%)	—	—	32 (21.2%)	5 (35.1%)	8 (5.3%)	42 (27.8%)	42 (27.8%)	NA	80 (52.9%)
Kapoor et al., 2018	16	2 (13%) ^+^infection 5/16 (31%)	—	—	5 (31%)	—	—	—	4 (25%)	6 (37%)	31(81%)
Miao et al., 2018	112	66 (58.9%)	—	—	23 (20.5%)	4 (3.6%)	—	—	19 (17%)	NA	NA
Qiaolei et al., 2018	174	—	92 (52.9%)	—	57 (32%) 33 (18.9%) ^+^infection	6 (3.4%)	—	—	24 (13.8%)	NA	102 (58.6%)
Zhou et al., 2018	205	119 (58%)	—	—	83 (40.5%)	14 (6.8%)	—	—	14 (6.8%)	89 (43.3%)	NA
Kumar et al. 2019	12	—	—	—	4 (33.3%)	7 (58.3%)	—	—	1 (8.3%)	NA	NA
Jumic et al., 2019	41	16 (54%)	—	—	22 (41%)	8 (19%)	9 (22%)	—	—	NA	16 (39%)
Birndt et al., 2020	137	48 (35%)	—	—	61 (44.5%)	13 (9.5%)	—		15 (10.9%)	27 (20.6%)	67 (51%)
Diack et al., 2020	26	11 (42.3%)	—	1 (3.8%)	12 (46.1%)	6 (23%)	—	—	2 (7.6%)	NA	19 (73%)
Pandey et al., 2020	41	15 (37%)	—	—	31 (76%)	9 (22%)	—	—	—	NA	22 (54%)
Bichon et al., 2021	260	28 (11%)	205 (79%)	—	—	—	—	—	27 (10%)	NA	147 (75%)

^+^For 2 patients, the outcome is not available; ^++^Meta-analysis, 24 studies published between Jan 1, 1974, and Sept 29, 2011, none of them separately listed in our table; ^+++^39 deaths among 60 patients followed-up.

**Table 2:** Laboratory findings.

Parameters (references), units	At admission	After 1 month	After 3 months	After 6 months	After 12 months
Leucocytes (3.00–10.5), g/L	3.97	4.07	4.21	3.35	3.99
Neutrophils (1.60–7.40), g/L	4.70	2.57	2.25	1.89	2.38
Lymphocytes (1.10–3.50), g/L	0.24	0.68	1.44	1.03	1.10
Hemoglobin (121–154), g/L	96	96	91	122	128
Platelet count (150–450), g/L	74	76	337	216	220
Ferritin (20–250), *μ*g/L	33649	49036	77	508^∗^	254
ASAT (<35), U/L	213	175	16	—	31
ALAT (<35), U/L	56	462	30	32	39
Alkaline phosphatase (35–110), U/L	180	211	63	—	121
GGT (<40), U/L	113	504	43	—	48
LDH (<250), U/L	999	838	262	191	158
Triglyceride (<1.70), mmol/L	2.55	—	—	—	—
D-dimer (<500), *μ*g/L	71270	3643	—	<155	—
Fibrinogen (1.80–4.00), g/L	3.12	1.91	3.27	—	3.35

^∗^Iron deficiency was diagnosed one month before, and this value was after iron infusion administration.

**Table 3:** Saint-Antoine score [23] calculated for this case (https//saintantoine.aphp.fr/score/).

Known underlying immunodepression	No
Maximal temperature (°C)	Strictly greater than 39.4
Hepatomegaly	No
Splenomegaly	Yes
Lower hemoglobin level	Strictly greater than 92 g/L
Lower leukocyte count	Less than or equal to 5 g/L
Lower platelet count	Less than or equal to 110 g/L
Higher ferritin level (ng/ml)	Strictly greater than 6000
Higher triglyceride level (mmol/l)	Between 1.5 and 4
Lower fibrinogen level (g/L)	Strictly greater than 2.5
Higher ASAT/ALAT level (UI/l)	Greater than or equal to 30
Hemophagocytosis features on the bone marrow aspirate	No
	Calculate
HScore	209
Probability of having HS (%)	92.87735027549559

**Table 4:** Performed investigations to exclude infections, malignancies, rheumatic conditions, and genetic mutations.

Causes	Investigations	Results
Infections	Blood culture	Sterile
	Lumbar puncture	Sterile
	Transthoracic echocardiography	No vegetations
	QuantiFERON test	Negative
	SARS-CoV-2 PCR, SARS-CoV-2 spike antibodies, influenza PCR	Negative
	Viral serology (EBV, CMV, HIV, HAV, HBV, HCV, HDV, HEV, HHV6, parvovirus B19, *Bartonella*, leptospirosis, toxoplasmosis, *Coxiella*)	Negative

Malignancies	Thorax X-ray, abdominal ultrasound, CT of the thorax, abdomen, and pelvis	No suspected lesions
	Bone marrow biopsy, skin biopsy, liver biopsy	No malignancy
	PET-CT	Metabolic active lesions:
		1. Right knee
		2. Colon ascendants and sigma
	Right knee MRI	Pigmented villonodular synovitis (PVNS), nonmalignant
	Gastroscopy and colonoscopy	No malignancy

Rheumatic conditions	ANA, Hep2 cytoplasmic, SsA, PR3-ANCA, MPO-ANCA	Negative
	Rheumatic disease symptoms	None

Genetic causes	Genes *PRF1*, *STX11*, *STXBP2*, *UNC13D*	No pathologic variant

Polymerase chain reaction (PCR), Epstein–Barr virus (EBV), cytomegalovirus (CMV), human immunodeficiency virus (HIV), hepatitis A virus (HAV), hepatitis B virus (HBV), hepatitis C virus (HCV), hepatitis B virus (HBV), hepatitis C virus (HCV), hepatitis D virus (HDV), hepatitis E virus (HEV), human herpes virus 6 (HHV6), computed tomography (CT), positron emission tomography-computed tomography (PEC-CT), magnetic resonance imaging (MRI), antinuclear antibodies (ANAs), antineutrophil cytoplasmic antibodies (ANCAs), myeloperoxidase (MPO), perforin 1 (PRF1), syntaxin 11 (STX11), syntaxin binding protein 2 (STXBP2).

## Data Availability

Data analyzed in this study were reanalysis of the existing data, which are openly available at locations cited in the reference section.
